# Stromal fibroblast activation and their potential association with uterine fibroids (Review)

**DOI:** 10.3892/ol.2014.2225

**Published:** 2014-06-04

**Authors:** LI-HUA ZHENG, FENG-FENG CAI, ISABELL GE, EWELINA BISKUP, ZHONG-PING CHENG

**Affiliations:** 1 Department of Gynaecology and Obstetrics, Yangpu District Central Hospital, Tongji University, Shanghai 200090, P.R. China; 2 Department of Breast Surgery, Yangpu Hospital, Tongji University, Shanghai 200090, P.R. China; 3 Faculty of Medicine, Heidelberg University, Heidelberg, Baden-Württemberg D-69120, Germany; 4 Department of Oncology, University Hospital of Basel, Basel-Stadt CH 4055, Switzerland

**Keywords:** fibroblasts, fibroblast activation, uterine fibroids, α-smooth muscle actin, transforming growth factor-β

## Abstract

Uterine fibroids are the most common type of benign, gynecologic neoplasm and are the primary indication for performance of a hysterectomy, accounting for >200,000 hysterectomies annually in the USA. At present, females are younger and exhibit larger leiomyomas at the time of diagnosis. Cancer-associated fibroblasts in tumor microenvironments have emerged as an important target for cancer therapy. Repeated stimulation by infectious or non-infectious agents in the uterine tissues, including inflammation, mechanical forces or hypoxia, stimulate the resident fibroblasts to undergo specific activation and, thus, are significant in tumorigenesis. Furthermore, complex signaling pathways regulate the mechanisms of fibroblastic activation. The current review focuses on the molecular mechanisms of fibroblastic activation and the potential association with uterine leiomyoma pathogenesis, enabling an integrated pathogenic analysis for review of the therapeutic options.

## 1. Introduction

Uterine leiomyomas, termed uterine fibroids, are benign smooth muscle tumors, which are enriched in the extracellular matrix (ECM). They are associated with the highest morbidity rate in female reproductive tract tumors (1), and females currently tend to present with larger leiomyomas and are younger at diagnosis (2). Myomas are the primary indication for performance of a hysterectomy, accounting for >200,000 hysterectomies annually in the USA (1). The most common symptoms of leiomyomas are heavy bleeding and pelvic pain, which are associated with infertility and adverse birth outcomes, including fetal mortality (3). Heavy menstrual bleeding may be severe enough to lead to anemia, which requires blood transfusions. Furthermore, females that are diagnosed with uterine leiomyomas account for >2.5-fold higher healthcare expenses compared with females without a leiomyoma diagnosis and additional work disability costs (2). Notably, fibroids are the leading indication for performance of a hysterectomy in the USA, however, little is known regarding their etiology or pathogenesis despite their particularly high prevalence and serious impact on the lives of females.

Tumorigenesis is a multistep process, which is considered to be analogous with Darwinian evolution, whereby genetic changes result in a growth advantage in a subset of cells and their subsequent progression from a normal to a malignant state (4). However, a tumor mass is not defined by tumor cells alone, it is defined as a tissue in which a tumor microenvironment (TME) prevails. Thus, in the past decade, the TME and its constituent ‘stromal’ cells have collectively gained prominence, and are currently being widely investigated (5,6). Previously, tumor-associated fibroblasts (TAF) were presumed to be passive structural elements, however, currently there is a growing awareness that TAF and the complex cellular TME may be involved in early tumor development (4,7,8). The phenomenon of tumor-associated desmoplasia, characterized by enhanced fibroblast accumulation and a modified, collagenized ECM, has been comprehensively reviewed in tumors exhibiting a marked desmoplastic reaction, including tumors of the pancreas, breast and gastrointestinal tract (7,8).

Myomas are firm, circumscribed masses. They possess a smooth muscle component and a significant ECM, which principally consists of fibroblasts, often termed myofibroblasts, which predominantly produce collagens type I and III (9). Myomas have been found to mimic the fibrotic process, and have been shown to specifically upregulate collagen types I and III (9–11). In addition, it has been proposed that the pathogenesis of myomas is comparable to an injury response (analogous to keloid development) following surgery (12). Mladenović-Mihailović *et al* (13) investigated the immunocytochemical characteristics of smooth muscle cells (SMCs) and connective tissue components of uterine submucosal myomas. They were found to consist of SMCs of the highly differentiated contractile and proliferate phenotypes [α-smooth muscle actin (SMA)-, desmin- and proliferating cell nuclear antigen-immunoreactivity], as well as connective tissue as a result of the synthetic activity of the fibroblasts. The two components markedly differ in their immunocytochemical characteristics from SMCs of the synthetic phenotype (13). Furthermore, Moore *et al* (9) revealed that human uterine leiomyoma-derived fibroblasts stimulate uterine leiomyoma cell proliferation and collagen type I production, as well as activate receptor tyrosine kinases and transforming growth factor (TGF)-β-receptor signaling in co-cultures. These findings indicate the importance of the interactions between fibroid tumor cells and ECM fibroblasts *in vivo*, as well as the role of growth factors and ECM proteins in the pathogenesis of uterine fibroids. Thus, it may be hypothesized that carcinoma-associated fibroblasts are important in the pathogenesis of myomas. The present review focuses predominantly on the overall activation of TAFs in the tumorigenesis of uterine fibroids.

### TAFs

Fibroblasts are the predominant source of the ECM in normal and tumor tissues, and are capable of producing several ECM-modulating factors (8). Due to the abundance of ECM often observed in fibroids, Moore *et al* (9) concluded that the interactions between leiomyoma SMCs and fibroblasts are important for the growth of such tumors as a result of their impact on the production of growth factors and ECM proteins. Tumor-associated ECM is an aberrant and complex meshwork of collagens, fibrillar glycoproteins and proteoglycans that determine abnormal tumor architecture. Furthermore, perturbations in the production, deposition and degradation of matrix components have been observed in numerous human tumors, including leiomyomas (9).

Quiescent fibroblasts, an arrested phenotype of TAF, are unable to promote the desmoplastic reaction of tumors during wound healing, tissue repair and scar-like pathogenesis unless they are activated or have differentiated into myofibroblasts. The present review focuses on the fibroblast activation pathway. Activated fibroblasts and myofibroblast cells that exhibit the appearance of fibroblasts, but express myocyte markers, including the unique marker fibroblast activation protein and α-SMA (the most reliable markers for the maturation of fibrocytes) are critical in the genesis of uterine tumor fibrosis during genital tract inflammation (4,14). One consistent phenotype of TAF, the myofibroblast, exhibits a muscle-like morphology and marked microfilamentous apparatus, resulting in a contractile profile. Once the fibroblasts are activated, TGF-β promotes mitogenesis and upregulates the synthesis of numerous components of ECM, leading to fibrosis.

## 2. TGF-β stimulate stromal fibroblasts

TGF-β is a multifunctional cytokine, which is important in embryonic development, and the regulation of repair and regeneration processes following tissue injury (15). This large superfamily of soluble factors includes three isoforms, TGF-β1, -β2 and -β3, which are encoded by three separate genes, but bind to the same high affinity receptor (16,17). Powell *et al* (18) reported that TGF-β1 is the isoform that is commonly upregulated in the presence of a tissue injury. It is secreted in a latent form following cleavage from a large pro-molecule. It binds non-covalently to the membrane-associated latency-associated peptide, which is formed from the cleavage fragments of the TGF-β1 precursor. This latent TGF-β1 is then stored on the cell surface or in the ECM, awaiting the conversion to active TGF-β1, via an unknown mechanism (19).

Feghali *et al* (16) reported that TGF-β is primarily produced by active T cells, platelets and monocytes in an anti-infection immunity milieu. At the site of injury, TGF-β, which is stored in platelets is released upon degranulation. Sarkar *et al* (20) also demonstrated that T cells, however, not tumor cells are a critical source of TGF-β1, which inhibits antitumor T cell responses and, thus, fosters tumor growth, which promotes tumor development. However, which cells are actually responsible for the chronicity of inflammation remains unclear. Immune cells may be activated by an unknown primary antigen or by the products of surrounding non-immune or mesenchymal cells activated by immune cells or self-derived cytokines. It is known that TGF-β attracts monocytes and other leukocytes to the inflammation site, thus participating in the initial step of chronic inflammation. Recently, a seventh hallmark, cancer-associated inflammation, was proposed by Colotta *et al* (21), two years following the hypothesis proposed by Wegienka *et al* (2) that leiomyomas are caused in part by a systemic immune milieu that is chronically inflammatory (22). Inflammation may be problematic if it is not well regulated, and thus a proper treatment for inflammation would substantially reduce the mortality and the therapy costs associated with these tumors.

The theory that injury or reproductive tract infections may trigger fibroid development was introduced many decades ago (22), however, it has not been adequately analyzed. In addition, Laughlin *et al* (23) indicated that certain pathogens do not remain latent in fibroid tissue and hypothesized that they may exhibit an acute ‘hit and run’ effect on tumor initiation or tumor growth, whereby having infected the tissue once, they may induce macrophage activity and immunocyte lethality (24). Innate immune responses initiate an anti-inflammatory process, starting with the recognition of mucopeptides and the activation of alternative complement pathways. Certain types of protein in the cell wall stimulate CD4^+^ T cells and produce large quantities of cytokines, including TGF-β. As a result of immunogenic variation and other forms of immune invasion, innate and adaptive immunity may fail to clear pathogens, which contribute to chronic inflammation and subsequent persistent and repeated infections.

The extracellular concentration of active TGF-β is primarily regulated by the conversion of latent TGF-β to active TGF-β. However, numerous studies have overlooked the activation process, possibly due to the complex biological nature of TGF-β. Mammalian TGF-β is secreted in a latent form that is composed of three proteins derived from two genes. One of the genes encodes for TGF-β and latency-associated peptide (LAP) (25). The mechanism of latent TGF-β activation is a topic of intense investigation and various details require investigation. Latent TGF-β binding protein is primarily involved in TGF-β localization by interacting with the local matrix during activation, whereby TGF-β is liberated from LAP and becomes activated (25). As soon as the repair is complete, TGF-β and ECM production is subsequently shut down by an unknown mechanism. The two functions are critical for maintaining homeostasis (15).

### Mechanism of fibroblast activation

**Figure 1:**
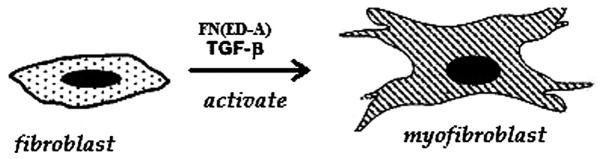
Fibroblast transformation. A schematic diagram that demonstrates the activation and stellate transformation of myofibroblasts, which requires the presence of matrix molecules, specifically the ED-A domain of fibronectin (7). The arrow represents activation. TGF-β, transforming growth factor; FN, fibronectin.

TGF-β appears to be the most important cytokine that activates the fibroblasts (25,26). Recently, it has been demonstrated that the activation of the myofibroblast requires the presence of matrix molecules, in particular, the ED-A (EIIIA) domain of fibronectin. Tissue injury results in the production of this specific ED-A domain splice variant of fibronectin. ED-A is the binding site for cell membranes and for other matrix molecules. Furthermore, it has been shown, in skin granulation tissue and hepatic models, that the fibronectin ED-A domain is necessary for TGF-β to trigger α-SMA expression and collagen secretion in the stellate transformation of myofibroblasts (18)(Fig. 1).

**Figure 2:**
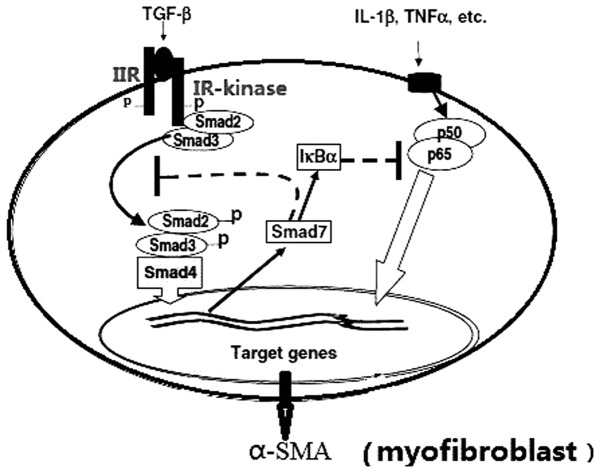
TGF-β activates fibroblasts. TGF-β binds to the specific TGF-β receptor and activates Smad2/3. The activated Smad2/3 associates with Smad4 and translocates to the nucleus to regulate gene expression, which is critical to fibrosis. Activation of Smad2/3 may also induce the expression of Smad7, which inhibits Smad activation and induces the expression of IκBα, an inhibitor of nuclear factor-κB, thereby inhibiting the inflammatory response. Dotted arrows represent an inhibitory interaction (26). TGF-β, transforming growth factor; IL-lβ, Interleukin-1β; TNF-α, tumor necrosis factor-α; α-SMA, α-smooth muscle actin.

Briefly, TGF-β initiates the cellular response by binding to its distinct TGF-β II receptor. The ligand binding cascade activates the TGF-β-RI kinase, which phosphorylates the receptor regulated Smads (R-Smads). The activated R-Smads form oligomeric complexes with the common Smad (Co-Smad; Fig. 2).

The oligomeric complexes then translocate into the nucleus, where they regulate the transcription of target genes by binding to DNA directly or indirectly via interaction with various cofactors (Fig. 2). TGF-β may also stimulate inhibitory Smads, which negatively regulate TGF-β signaling transduction (27).

R-Smads, including Smad2, -3 and the Co-Smad (Smad4), contain conserved amino- and carboxyl-terminal mad-homologies (MH) 1 and 2, respectively, which flank a more divergent middle linker region (26,27). The MH1 domain is the functional unit that binds DNA directly to regulate gene transcription, whereas the MH2 domain contains the SSXS phosphorylation site (Ser-465/Ser-467), which is typically phosphorylated by the TGF-β receptor I serine kinase (28). TGF-β induced accumulation of ECM predominantly occurs via the Smad3-associated downregulation of matrix metalloproteinase-1 and positive regulation of tissue inhibitor of matrix metalloproteinases-1. Smad3 binds directly to DNA, whereas Smad2 binds to coactivators or repressors to regulate its target gene activities. As a result of Smad signals that promote the expression α-SMA, the fibroblasts are activated and differentiated.

## 3. Mechanical forces activate fibroblasts

A study by Petersen *et al* (29) presented a novel insight with regards to the effects of mechanical loading on the production and remodeling of ECM components, as well as the impact of the altered mechanical cell environment on these processes. The theory of cellular mechanotransduction has been proposed in recent years, which indicates that mechanical and chemical signals may interact to control cell growth, differentiation, movement and death. Ingber (30) reported that cytoskeletal tension affects the integrity of the shape and function of cells, analogous to the tent model (31). The association between cell mechanics and biochemistry is dependent on integrins, discrete focal adhesions, ECM substrates and the cytoskeleton; therefore, controlling cell shape is important in managing the structural and informational complexity of living cells.

Connective tissues do not passively bear the stress resulting from gravity, compression and muscle-generated forces. They interplay with these factors dynamically by modifying their composition and mechanical properties. At the cellular level, mechanical signals influence cell morphology, cytoskeletal reorganization, cell survival, cell differentiation and gene expression (32). Similarly, cells contain a set of specific structures, the cytoskeleton, which is capable of generating forces and bearing elastic deformation (33).

Mechanical forces include fluid flow, direct compression and tensile stress. They are essential regulators of tissue homeostasis and are essential for the correct functioning of connective tissues, since these are subjected to the greatest levels of stress in an organism (34). All adherent cells, including endothelial cells, fibroblasts and myofibroblasts sense tension, which originates from the environment. Tension is transmitted via cell-ECM contact, which leads to the reorganization of the cytoskeleton and the elicitation of specific signals that modulate gene expression. Cells are continuously recognizing alterations in mechanical forces and their functions are adapted according to the biological requirements. When mechanical tension is removed (30), tissues undergo atrophy, which demonstrates the importance of mechanical signals in maintaining the proper functioning of the organism. Malik *et al* (36) investigated the altered mechanical homeostasis in uterine leiomyomas, which had been exposed to increased mechanical stress. Structural and biochemical features were observed to be consistent with the activation of solid-state signaling. Thus, stress may be a contributing factor to leiomyoma growth.

As previously stated, cells firmly attach to ECM structures via matrix adhesions. These include focal complexes, and focal and fibrillar adhesions. The major structures that are required to form such matrix contacts are the integrin receptors, which directly connect the ECM structures to the intracellular cytoskeleton network (36). Mechanical forces act on focal adhesions, resulting in further structural maturation. The mechanisms by which fibroblasts transmit mechanical signals remain unclear, however, they may involve stretch-activated ion channels, direct interactions between structural and signaling components or the activation of small guanosine triphosphatases (GTPases).

As previously described, numerous cooperative interactions exist between integrins and growth factor signaling. Specifically, fibroblast to myofibroblast conversion and α-SMA expression depend on a combination of mechanical tension and TGF-β activity. Thus, in scarring, the generated tensions may induce myofibroblast formation, resulting in a self-perpetuating loop (37). A similar autocrine loop is observed in the induction of collagen synthesis in fibroblasts by mechanical tension, whereby TGF-β is induced by tension, which in turn activates collagen synthesis via the usual signaling pathways.

The formation of stress fibers and the neo-expression of α-SMA is a hallmark of fibroblast to myofibroblast differentiation. This change is a significant event in the development of fibro-contractive diseases and in wound granulation tissue contraction. The incorporation of the SMA isoform into stress fibers confers a high contractile activity to myofibroblasts. This is subsequently transmitted to the ECM at sites of specialized adhesions, termed ‘fibronexus’ in tissue and ‘supermature focal adhesions’ in two-dimensional cell cultures (38). In addition, Hinz (39) proposed that myofibroblast differentiation requires a mechanically restrained environment in conjunction with the action of growth factors (TGF-β) and specialized matrix molecules (ED-A splice variant of fibronectin). Myofibroblast adhesions sense matrix stress and transmit contractile force to the extracellular environment, in addition to producing the high intracellular tension that is required for myofibroblast development (39).

This clearly demonstrates that mechanical tension, which is generated during wound contraction or scar formation, may modulate the gene expression of fibroblasts and myofibroblasts embedded into this tissue at different molecular levels. Tension directly modifies gene transcription via the induction of integrin signaling, which affects small GTPases or induces/inhibits growth factor signaling, which subsequently indirectly affects ECM protein synthesis in the fibroblasts/myofibroblasts (36). Via a combination of these mechanisms, mechanical tension induces an activated, contractile fibroblast phenotype, which is characterized by high levels of ECM protein synthesis and fibrogenic cytokine production, as well as low protease activity.

### Signaling mechanisms potentially involved in the regulation of actin genes by mechanical stress

Previous data (36–40) indicates that mechanical signals specifically regulate the synthesis and degradation of various ECM components. The forces exerted by the cells themselves are generated by the cytoskeleton and are measurable. In electrically excitable cells, stretch-sensitive cation channels are important for sensing strain (41). Therefore, it is likely that in connective tissue cells, such as fibroblasts, cell-matrix adhesions are the functional strain gauges that sense the mechanical properties of the ECM as well as the environmental changes. Focal adhesions, evolving from focal complexes (small dot-like adhesion sites), undergo further structural maturation depending on externally applied or cytoskeletal forces (33). Furthermore, integrin activation triggers intracellular signaling events. Mechanical stress applied directly to integrin ligands elicits chemical responses inside the cell cascade, including the assembly and growth of focal contacts.

**Figure 3:**
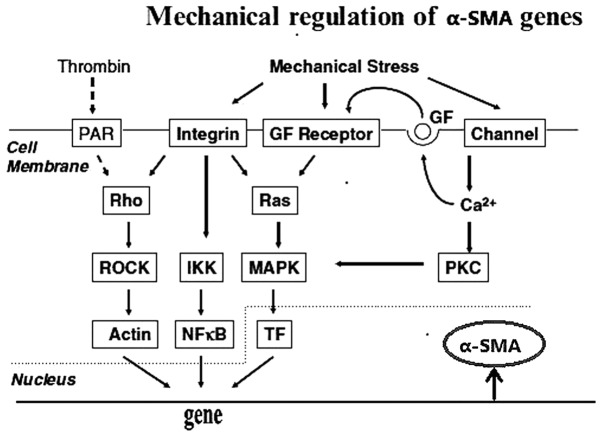
Mechanical forces activate fibroblasts. Intergrins and stretch-activated ion channels act as receptors and mechanical forces activate the Rho family, IKK, mitogen-activated protein kinase p38 and PKC via downstream elements, which regulates α-SMA. Dotted lines represent inhibitory interactions (31). α-SMA, α-smooth muscle actin; PAR, protease activated receptor; GF, growth factor; ROCK, Rho kinase; IKK, IκB kinase; MAPK, mitogen-activated protein kinase; NF-κβ, nuclear factor-κβ; TF, transcription factor; PKC, protein kinase C.

The earliest responses to mechanical stimulation are recorded at the cell-ECM adhesion level. These include the opening of stretch-activated ion channels, release of soluble mediators, phosphorylation of focal adhesion-associated kinases (for example, focal adhesion kinase, Src and integrin-linked kinase), activation of small GTPases (including RhoA), increased phosphatidyl inositol metabolism and generation of reactive oxygen species (42,43). Multiple intracellular signaling pathways are subsequently triggered, including those involving mitogen-activated protein kinase (MAPK), protein kinase C and nuclear factor κB (44). Overall, the cascades lead to the regulation of the target gene of α-SMA at the gene transcription level. There are at least three regulatory mechanisms that use the abovementioned pathways, subsequently leading to the activation of fibroblasts (Fig. 3).

## 4. Hypoxia

Kawaguchi *et al* (45) demonstrated that myocardial ischemia/reperfusion injury triggers the activation of the inflammasome in fibroblasts. These observations revealed that chronic and sustained hypoxia, loss of stromal fibroblast, caveolin-1 (as a biomarker for chronic hypoxia), oxidative stress and autophagy (46) induce a proinflammatory and profibrotic microenvironment in rat pulmonary arteries (47). In addition, hypoxia-induced proteomic changes in neoplastic and stromal cells influence tumor propagation (44). Hypoxia-mediated malignant progression has been debated as a leading factor that leads to multidrug resistance. In previous animal models (41), the earliest and most evident structural changes following hypoxic exposure were identified in the adventitial compartment of the vascular walls. Furthermore, resident adventitial fibroblasts have been shown to exhibit early and sustained increases in proliferation that exceed those observed in endothelial or SMCs.

### Hypoxia-induced proliferation is dependent on MAPKs

**Figure 4:**
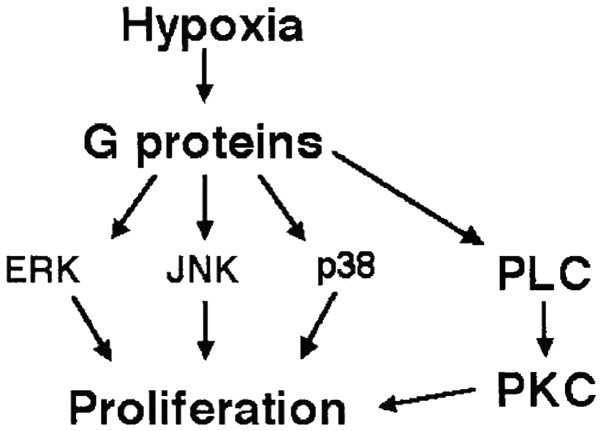
Hypoxia-induced activation of fibroblasts. The hypoxia-induced proliferation of fibroblasts is transduced through G protein-mediated activation of mitogen-activated protein kinases (46). ERK, extracellular signal-regulated kinase; JNK, c-Jun N-terminal kinase; PLC, phospholipase C; PKC, protein kinase C.

The increased expression of α-SMA-positive cells (myofibroblasts) has also been observed in neonatal calves following acute hypoxic exposure (48). Hypoxia has been reported to activate MAPK signaling pathways in numerous cell types, although very few of those cells demonstrate a proliferative response under hypoxic conditions. In fibroblasts, a hypoxia-induced transient activation of extracellular signal-regulated kinases 1/2 and c-Jun N-terminal kinase and a biphasic activation of p38 MAPK was observed (Fig. 4).

### Activation of fibroblasts induced by hypoxia

Based on observations demonstrating that stimuli, including sheer stress, pH and osmolality, may activate Gi-proteins with subsequent activation of MAPK signaling, Gerasimovskaya *et al* (49) proposed that hypoxia in the absence of exogenous ligands directly activates Gi/o-mediated signaling. In addition, hypoxia itself may act as a growth-promoting stimulus for bovine neonatal adventitial fibroblasts via Gi/o (and possibly Gq)-mediated activation of a complex network of MAPKs (48).

Furthermore, hypoxia has been shown to activate G-protein-coupled receptor signaling pathways. Stenmark *et al* (48) hypothesized that hypoxia may act as a stimulus for the induction of the differentiation of fibroblasts into myofibroblasts. Hypoxia was found to induce a marked increase in α-actin protein in fibroblast subpopulations of neonatal bovine PA adventitial fibroblasts (50). To investigate the underlying molecular mechanisms, fibroblasts were transiently transfected with a luciferase-tagged α-SMA promoter and subsequently exposed to hypoxia. Hypoxia induced an increase in α-SMA promoter activity and this induction of α-SMA promoter activity was observed to be largely independent of TGF-β activity. Thus, these results indicate that hypoxia-induced α-SMA expression in fibroblasts is mediated by Gi-proteins (Fig. 4).

### Synergy between hypoxia and adenosine receptors

Chronic inflammatory diseases are commonly associated with hypoxia, which has been shown to be a powerful stimulus for gene expression and cell differentiation (51,52). Hypoxia and the activation of A_2B_ adenosine receptors act synergistically to promote the release of interleukin (IL)-6. Zhong *et al* (54) demonstrated that the activation of A_2B_ adenosine receptors increased the release of IL-6. This proinflammatory cytokine, which mediates inflammation, is exhibited at elevated concentrations in the lung of individuals with asthma and induces the differentiation of human lung fibroblasts into myofibroblasts. The induction of α-SMA expression (by adenosine) is an essential feature during this process (53). At present, the cellular source of the adenosine is unknown. Under hypoxia, the effect of adenosine on α-SMA expression is not completely blocked by anti-IL-6. There may be additional factors, including platelet-activating factor and platelet-derived growth factor, which also contribute to the synergistic effect of adenosine and hypoxia on α-SMA. Notably, IL-6 was demonstrated to inhibit the proliferation of normal fibroblasts and induce proliferation of idiopathic pulmonary fibrosis fibroblasts (54).

The initial evidence regarding the critical effect of hypoxia on TAFs (55) may lead to further investigation into the regulatory mechanisms, which are relevant to fibroblasts in an oxygen-deficient (tumor) micro-milieu, in order to establish novel fibroblast-based therapeutic designs.

## 5. Inactivation of fibroblasts

### Novel role of thrombospondin-1 (TSP-1)

Wu *et al* (56) demonstrated that a downregulation of TSP-1 during cervical carcinogenesis was accompanied by the upregulation of stromal markers, α-SMA and desmin. The transfection of NIH/3T3 cells with TSP-1 and purified TSP-1 did not alter the protein levels of α-SMA and desmin, however, significantly inhibited matrix metalloprotease-2 activity.

TSP-1 expression was higher in the tumors or tumor-associated stroma when compared with the expression in normal epithelial (57). TSP-1 inhibited fibroblast invasion regardless of the presence of TGF-β, however, a higher dose of TSP-1 was required for the complete inhibition of TGF-β-treated NIH/3T3 cells. The complexity and duality of the functions of TSP-1 and TGF-β may result from the ability to suppress tumor cell proliferation at the early stage, whilst enhancing the host stroma reaction at the later stages (58). Further investigation is required to elucidate the dynamic interaction that exists between TSP-1 and TGF-β in the regulation of cervical cancer growth. Notably, TSP-1-mediated inhibition was only demonstrated in the fibroblasts with manipulated TSP-1 expression, however, not in the tumor cells. TSP-1 exerts its effects, including the inhibition of fibroblast migration, decreasing the recruitment of inflammatory cells, induction of endothelial cell apoptosis or the activation of SMC proliferation, in multiple types of stromal cells (59).

The effects of TSP-1 on tumorigenesis differ markedly from those on stromal cells. This indicates that TSP-1 exerts various biological functions in different cell types. For example, a switch in angiogenesis phenotype during the transition from low- to high-grade squamous intraepithelial lesion occurs partly due to the downregulation of TSP-1 (57). The genetic manipulation of TSP-1 expression levels in cells revealed that TSP-1-mediated inhibition of stromal reactions is primarily due to the inhibition of activated fibroblast migration and invasion, rather than a direct effect on stromal marker expression (60).

Unlike TSP-1, secreted protein, acidic and rich in cysteine (SPARC), was shown to inhibit fibroblast activation by blocking α-SMA overexpression (61,62). Although SPARC and TSP-1 are matricellular proteins, which inhibit angiogenesis and interfere with ECM organization (63), TSP-1 inhibits stromal reactions via a mechanism that is distinct from SPARC.

### The function of SPARC

SPARC, also termed osteonectin or BM-40, is a Ca^2+^-binding matricellular glycoprotein involved in wound healing, neoplasia and the mediation of cell-matrix interactions. Chlenski *et al* (62) reported that in addition to stromal formation enhancement, SPARC prevented fibroblast activation in 293 xenografts, indicating that the anticancer effects of SPARC may be due to the formation of tumor stroma, which do not support tumor growth (56).

Interactions between tumor and inflammatory cells determine tumor progression or regression via numerous mechanisms, including stromal formation, angiogenesis, adhesion and cell migration. A cytokine- and chemokine-rich milieu, together with other factors contributes to tissue remodeling. Emerging evidence proposes that SPARC produced by host leukocytes, rather than the tumor, determines the assembly and function of tumor-associated stroma via collagen type IV organization (64).

The actin cytoskeleton of animal cells maintains the cellular shape and is significant in cell motility. Rho and Rac (two members of the Ras-associated superfamily of small GTPases) and Cdc42 (another member of the Rho family), regulate the polymerization of actin to produce stress fibers or lamellipodia, respectively; the Rho family of small GTPases controls stress fiber formation. In particular, the activation of the Rho-Rac-Cdc42 signaling pathway results in stress fiber assembly via the activation of actomyosin contractility and suppression of the actin-severing activity of cofilin. Overexpression of SPARC in DAOY medulloblastoma cells inhibits Rho-Rac-Cdc42 GTPase activity and thus contributes to the inactivation of fibroblasts (65).

## 6. Conclusion

Uterine fibroids, the benign smooth muscle tumors originating from the myometrium, are responsible for the incidence of morbidity in a large number of females. Although their exact pathogenesis remains unknown, there is substantial evidence, which indicates that myomas consist of large quantities of uterine leiomyoma cells and fibroblasts. The present review predominantly focuses on a novel mechanism of fibroblast activation and its potential association with uterine fibroids. Thus, such novel insights may be considered useful for further investigation and future non-surgical treatment of leiomyomas.
